# Cell-Type Specific Distribution of T-Type Calcium Currents in Lamina II Neurons of the Rat Spinal Cord

**DOI:** 10.3389/fncel.2018.00370

**Published:** 2018-10-17

**Authors:** Jing Wu, Sicong Peng, Linghui Xiao, Xiaoe Cheng, Haixia Kuang, Mengye Zhu, Daying Zhang, Changyu Jiang, Tao Liu

**Affiliations:** ^1^Department of Pediatrics, The First Affiliated Hospital of Nanchang University, Nanchang, China; ^2^Department of Anesthesiology, The First Affiliated Hospital of Nanchang University, Nanchang, China; ^3^Department of Pain Clinic, The First Affiliated Hospital of Nanchang University, Nanchang, China; ^4^Jisheng Han Academician Workstation for Pain Medicine, Nanshan Hospital, Shenzhen, China; ^5^Center for Experimental Medicine, The First Affiliated Hospital of Nanchang University, Nanchang, China

**Keywords:** T-type calcium channel, spinal dorsal horn, lamina II neuron, T-type current, whole-cell patch-clamp recording

## Abstract

Spinal lamina II (substantia gelatinosa, SG) neurons integrate nociceptive information from the primary afferents and are classified according to electrophysiological (tonic firing, delayed firing, single spike, initial burst, phasic firing, gap firing and reluctant firing) or morphological (islet, central, vertical, radial and unclassified) criteria. T-type calcium (Cav3) channels play an essential role in the central mechanism of pathological pain, but the electrophysiological properties and the cell-type specific distribution of T-type channels in SG neurons have not been fully elucidated. To investigate the electrophysiological and morphological features of T-type channel-expressing or -lacking neurons, voltage- and current-clamp recordings were performed on either transverse or parasagittal spinal cord slices. Recording made in transverse spinal cord slices showed that an inward current (*I*_T_) was observed in 44.5% of the SG neurons that was fully blocked by Ni^2+^ and TTA-A2. The amplitude of *I*_T_ depended on the magnitude and the duration of hyperpolarization pre-pulse. The voltage for eliciting and maximizing *I*_T_ were −70 mV and −35 mV, respectively. In addition, we found that most of the *I*_T_-expressing neurons are tonic firing neurons and exhibit more negative action potential (AP) threshold and smaller difference of AP threshold and resting membrane potential (RMP) than those neurons lacking *I*_T_. Consistently, a specific T-type calcium channel blocker TTA-P2 increased the AP threshold and enlarged the difference between AP threshold and membrane potential (I_hold_ = 0). Meanwhile, the morphological analysis indicated that most of the *I*_T_-expressing neurons are islet neurons. In conclusion, we identify a cell-type specific distribution and the function of T-type channels in SG neurons. These findings might provide new insights into the mechanisms underlying the contribution of T-type channels in sensory transmission.

## Introduction

T-type calcium channels are low-voltage activated (LVA) calcium channels, consisting of three isoforms: Cav3.1, Cav3.2 and Cav3.3 (Bourinet et al., 2016). They are broadly distributed in vertebrates, including the central and peripheral nervous system, heart, smooth muscle and so on Iftinca (2011). In the nervous system, T-type channels have been implicated in the modulation of neuronal excitability, thus they are linked to the pathogenesis of various neurological disorders, including epilepsy, autism and chronic pain (Zamponi, 2016). Abundant expression of T-type channels has been found in pain-processing pathways, including dorsal root ganglion (DRG) and superficial spinal dorsal horn (SDH; François et al., 2015). Changes in the expression and function of T-type channels can cause an increase in the magnitude of responses to nociceptive stimulus, resulting in pain facilitation (Bourinet et al., 2005; Latham et al., 2009; Jacus et al., 2012; Rose et al., 2013; Stemkowski et al., 2016; Lai et al., 2017). In contrast, blockade of T-type channels has been proved to be efficient for pain relief (Garcia-Caballero et al., 2014; Snutch and Zamponi, 2017).

Spinal lamina II, also named substantia gelatinosa (SG), is an important primary relay station for the nociceptive signals conducted by thinly myelinated Aδ and unmyelinated C fibers (Kuner, 2010; Todd, 2010; Kuner and Flor, 2016). After modulated in SG, these signals are further transmitted to distinct projection regions in the brain. Therefore, the net output of the SG neurons plays an essential role in pain perception. Lamina II consists of excitatory and inhibitory interneurons, which can be further classified by different properties of electrophysiology and morphology (Todd, 2010). Although several studies have revealed that T-type currents could be recorded in SDH neurons (Ryu and Randic, 1990; Ku and Schneider, 2011), their exact expression pattern and electrophysiological properties in SG neurons are largely unknown.

In the present study, we detected a T-type channel-induced current (*I*_T_) in 212 out of 487 SG neurons (43.5%). Moreover, we found that the excitability of *I*_T_-expressing neurons is higher than that of *I*_T_-lacking neurons. This difference could be imitated by a selective T-type calcium channel blocker TTA-P2. Besides, we identified that most of the T-type channel-expressing neurons belong to the islet neurons (41.2%) or the tonic-firing neurons (79.4%). These results might provide new insights into the function of T-type channels in SDH.

## Materials and Methods

### Animals

Male and female Sprague-Dawley (SD) rats (3–5-week-old) were obtained from the Animal Center of Nanchang University, which were housed in a 12:12-h light-dark cycle. Food and water were available *ad libitum*. All efforts were made to minimize the animal suffering and to reduce the number of animals used in the experiment. This study was carried out in accordance with the recommendations of “protocol for the use of nonhuman vertebrates in research of the Institutional Animal Care and Use Committee of Nanchang University.”

### Spinal Cord Slice Preparation

Spinal cord slices were prepared as our previous studies (Liu et al., 2015; Hu et al., 2016). Briefly, animals were deeply anesthetized with urethane (1.5 g/kg, i.p.) and were transcardially perfused with ice-cold carbonated (95% O_2_ and 5% CO_2_) dissection solution containing (in mM): 240 sucrose, 2.5 KCl, 3.5 MgCl_2_, 0.5 CaCl_2_, 1.25 NaH_2_PO_4_, 0.4 ascorbic acid, 2 sodium pyruvate, 25 NaHCO_3_ and 1 kynurenic acid. The lumbosacral sections were quickly extracted and immersed in the same solution. After removing the pia-arachnoid membrane and the roots, the enlargement part of spinal cord was mounted on a vibratome (VT1000S; Leica, Nussloch, Germany) cutting stage. Transverse (450 μm) or parasagittal slices (300 μm) were cut and moved to an incubator, which was filled with carbonated artificial cerebral spinal fluid solution (aCSF) at 32°C for at least 30 min. The aCSF contained (in mM): 117 NaCl, 3.6 KCl, 2.5 CaCl_2_, 1.2 MgCl_2_, 1.2 NaH_2_PO_4_, 25 NaHCO_3_, 11 D-glucose, and 2 sodium pyruvate.

### Whole-Cell Patch-Clamp Recordings

Whole-cell voltage- and current-clamp recordings from SG neurons were obtained as previously described (Liu et al., 2015). In brief, one spinal cord slice was moved to the recording chamber and continuously perfused with aCSF at room temperature with a perfusion rate of 2–4 ml/min. Recording electrodes were pulled from borosilicate glass (1.5 mm OD, 1.12 mm ID; World Precision Instruments, Sarasota, FL, USA) with a micropipette puller (P-97; Sutter Instrument, Novato, CA, USA). The typical resistance of the pipette was 3–6 MΩ when filled with intracellular solution. The intracellular solution for recording T-type current in transverse slices contained the following (in mM): 92 CsMeSO4, 43 CsCl, 10 phosphocreatine, 0.5 EGTA, 10 HEPES, 4 Mg-ATP, 0.3 Li-GTP and 5 tetraethylammonium (TEA)-Cl (pH = 7.2, adjusted with CsOH, 300 mOsm). The intracellular solution for recording T-type calcium current and intrinsic membrane properties in parasagittal slices was (in mM): 130 K-gluconate, 5 KCl, 10 phosphocreatine, 0.5 EGTA, 10 HEPES, 0.3 Li-GTP, 4 Mg-ATP (pH = 7.3, adjusted with KOH, 295 mOsm). Signals were amplified with an EPC-10 amplifier and Patchmaster software (HEKA Electronik, Lambrecht, Germany). The SG neurons were visualized with a CCD camera, high-resolution water-immersion objective, and infrared differential interference contrast optics (IR-1000; Dage, Michigan City, IN, USA). Series resistances were typically measured between 10 MΩ and 30 MΩ and were monitored throughout the recording period. Data were excluded if the series resistance changed by >20%.

### Morphological Reconstruction

For morphological experiments, K^+^-based intracellular solution containing 0.05% neurobiotin 488 was used. After maintaining stable electrophysiological recording for at least 20 min, the slices were transferred to a container filled with 4% PFA at RT for 1 h and then at 4°C overnight for fixation. Neuronal 3D images were reconstructed with a Zeiss LSM 700 confocal microscope at a condition of 20× magnification, 1.0–1.5 zoom, and 1.5-μm stack.

### Chemicals

TTA-A2 and TTA-P2 were obtained from Alomone Labs. Tetrodotoxin (TTX) was obtained from Tocris Bioscience (Bristol, UK). All the other drugs for electrophysiological experiment were obtained from Sigma-Aldrich (St. Louis, MO, USA). All chemicals were dissolved in distilled water except that TTA-A2 and TTA-P2 was dissolved in DMSO and were stored at −20°C unless otherwise mentioned.

### Statistical Analysis

Curve fitting was established by GraphPad Prism 7.0 (GraphPad Software, La Jolla, CA, USA). Statistical analysis was performed by SPSS version 17.0 (SPSS Inc., Chicago, IL, USA). Shapiro-Wilk test was used to assess the normal distribution of data. Levene test was used to test the homogeneity of variance. The comparison of two groups was determined by paired or unpaired student’s *t*-test. The comparison of multiple groups was assessed by one-way or two-way repeated measure (RM) analysis of variance (ANOVA). Bonferroni or Dunnett T3 *post hoc* test was used following one-way ANOVA. Sidak’s *post hoc* test was used following two-way RM ANOVA if the interaction between factors was significant. All data are given as the mean ± SEM. Significance was set at *p* < 0.05.

## Results

### Isolation of T-Type Calcium Currents in SG Neurons

**Figure 1:**
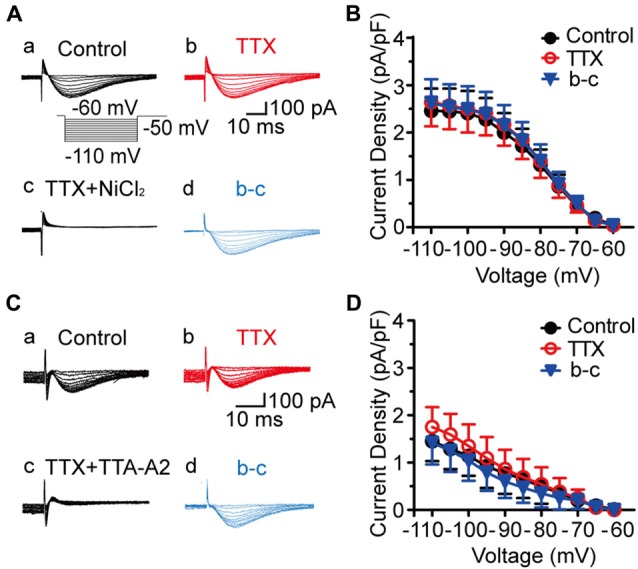
Isolation of T-type current in substantia gelatinosa (SG) neurons from the rat transverse spinal cord slices. **(A)** Representative families of current traces in control **(Aa)**, with 0.5 μM tetrodotoxin (TTX; **Ab**), 1 mM NiCl_2_ **(Ac)**, and that of the digital subtraction of **(Ab–Ad)**. The inset shows the voltage-clamp protocol. **(B)** Plots of T-type current density against the test potentials in various groups showing in **Aa**, **Ab**, and **Ad** (*n* = 19 cells in four rats). **(C)** Representative families of current traces in control **(Ca)**, with 0.5 μM TTX **(Cb)**, 40 μM TTA-A2 **(Cc)**, and that of the digital subtraction of **(Cb–Cd)**. **(D)** Plots of T-type current density against the membrane potentials in various groups showing in **Ca**, **Cb** and **Cd** (*n* = 7 cells in three rats).

To isolate the T-type currents, we used an intracellular solution containing 135 mM Cs^+^ and 5 mM TEA to block K^+^ currents. As shown in Figure 1Aa, voltage-dependent inward currents were evoked by a series of hyperpolarizing voltage steps from −60 mV to −110 mV in 5-mV decrements (500-ms duration) before stepping to −50 mV for 200 ms. These currents were detected in 178 out of 400 SG neurons from the transverse spinal cord slices. To assess the involvement of T-type channels in these inward currents, we first perfused the slice with TTX for 5 min (Figure 1Ab), and then added with 1 mM NiCl_2_ (Figure 1Ac) for 15 min. Under these conditions, NiCl_2_ completely blocked the inward currents. Net T-type currents were obtained by digital subtraction of the inward currents in the absence and presence of NiCl_2_ (Figure 1Ad). The current density, which was calculated by dividing the peak amplitude of T-type current by the cell capacitance, was not significantly different among those recorded in the conditions of control, TTX and digital subtraction of NiCl_2_ (*p* > 0.05; *n* = 19 cells in four rats; Figure 1B). As NiCl_2_ might have other non-specific actions, we repeated these experiments in the presence of a more specific T-type channel blocker, TTA-A2 (Figure 1C). Similar results were obtained under the treatment of TTA-A2 (40 μM), as the current density was not significantly changed before and after digital subtraction of TTA-A2 (*p* > 0.05; *n* = 7 cells in three rats; Figure 1D). Moreover, the amplitudes of the inward currents were not apparently different in the absence and presence of TTX. Together, these data suggested that the inward currents we recorded in SG neurons are T-type channel-mediated currents.

### Effect of T-Type Channel Blockers on SG Neurons

**Figure 2:**
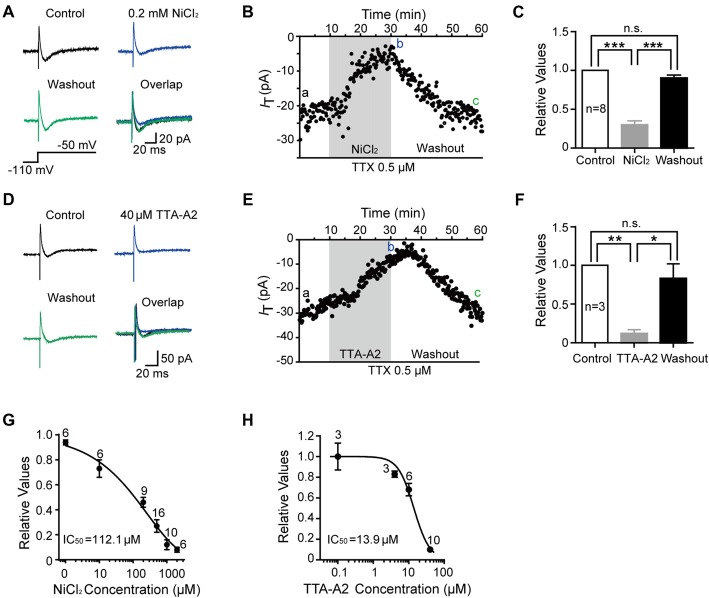
Pharmacological properties of T-type current in SG neurons. **(A)** Representative traces of T-type current in control, with 0.2 mM NiCl_2_, after washout of NiCl_2_, and the superimposed traces. In this figure, all the T-type current was activated every 10 s by a 200-ms voltage step to −50 mV from a holding potential of −110 mV for 500 ms (inset). **(B)** Time course of a representative experiment during application of NiCl_2_ that shown in panel **(A)**. The amplitude of T-type current is depicted over time. **(C)** Summary of the blockade of T-type current by NiCl_2_. **(D)** Representative traces of T-type current in control, with 40 μM TTA-A2, after washout of TTA-A2, and the superimposed traces. **(E)** Time course of a representative experiment during application of TTA-A2 that shown in panel **(D)**. The amplitude of T-type current is depicted over time. **(F)** Summary of the blockade of T-type current by TTA-A2. **(G,H)** Concentration-response relationship for NiCl_2_ and TTA-A2. Concentration-response curve was fitted with the Hill equation as follows: *y* = *I*_max_/(1 + IC_50_/x), in which *I*_max_ represents the maximal current amplitude, IC_50_ is the half-maximal inhibitory concentration, and *x* is the concentration. *P < 0.05, **P < 0.01, ***P < 0.001, ns, not significant.

We then investigated the dose-dependent effects of NiCl_2_ and TTA-A2 on the T-type current. As shown in Figures 2A,B, the amplitude of T-type current gradually decreased to 29.6 ± 4.6% of control (41.6 ± 8.6 pA; *p* < 0.001; *n* = 8 cells in five rats; Figure 2C) with bath application of NiCl_2_ (0.2 mM), reaching the peak at about 15–20 min. This effect was reversible since the amplitude of T-type current gradually recovered to 89.6 ± 3.9% of control after 30-min washout with aCSF (36.8 ± 7.4 pA; *p* = 0.063; *n* = 8 cells in five rats; Figure 2C). Perfusion with TTA-A2 (40 μM) also resulted in a decrease in the amplitude of T-type current to 12.1 ± 4.7% of control (153.9 ± 68.1 pA; *p* = 0.004; *n* = 3 cells in two rats; Figures 2D–F). After 30-min washout, the T-type current recovered to 83.0 ± 18.7% of control (123.04 ± 70.45 pA; *p* = 0.953; *n* = 3 cells in two rats). The half maximal inhibitory concentration (IC_50_) for NiCl_2_ and TTA-A2 were 112.1 μM and 13.9 μM, respectively (Figures 2G,H). In addition, we also observed an inhibitory effect of mibefradil (10 μM, 20 min) on the T current. Unlike NiCl_2_ and TTA-A2, the effect of mibefradil was irreversible (data not shown). These data suggested that various T-type channel blockers exhibit different patterns of effect on the inhibition of T-type current.

### Electrophysiological Features of T-Type Current in SG Neurons

**Figure 3:**
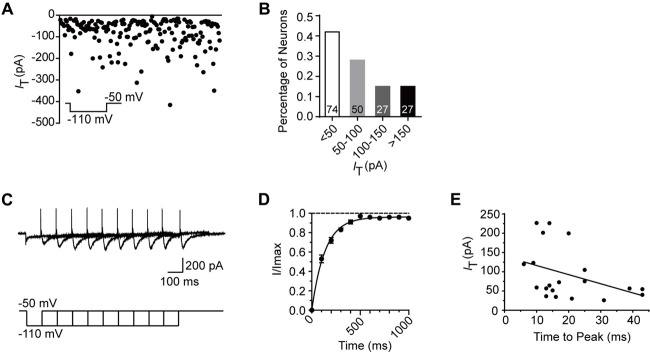
Electrophysiological properties of T-type current in SG neurons. **(A)** Plots of peak amplitude of T-type current in all the neurons examined from transverse slices (*n* = 195). The inset shows the voltage-clamp protocol. Inset: T-type current was activated every 10 s by a 200-ms voltage step to −50 mV from a holding potential of −110 mV for 500 ms. **(B)** Columns of various amplitude of T-type currents in fractions of the total neurons. The numbers in the column indicate the neurons examined. **(C)** Representative superimposed traces showing currents elicited by voltage steps to −110 mV of increasing duration. Lower panel shows the voltage-clamp protocol. **(D)** Recovery from the inactivation. As presented for plots of the relative amplitude of T-type current against the duration of the hyperpolarization which preceded it, fitting with a single exponential equation: *I*_(t)_ = *I*_T_ × exp(−*t*/τ), where *I*_(t)_ is the amplitude of the current at time t, *I*_T_ represents the amplitude of current, and τ is the time constant. Amplitude values were normalized relative to the maximum amplitude evoked following a 1 s hyperpolarization. **(E)** Plots of the time to peak vs. the amplitude of T-type current at the duration of 1 s.

To better understand the role of T-type current in the modulation of SG neurons, we next investigated the electrophysiological properties of this current in detail from transverse slices. The amplitude of T-type current was found to be varied from 6.1 pA to 415.4 pA (*n* = 178 cells in 60 rats), with the mean amplitude of 84.8 ± 5.3 pA at the holding potential of −110 mV (Figure 3A). The fractions were 42%, 28%, 15% and 15% in those amplitude <50 pA, 50–100 pA, 100–150 pA and >150 pA neurons, respectively (Figure 3B). Recovery from the inactivation was studied by voltage steps to −110 mV of increasing durations ranging from 100 ms to 1000 ms in 100-ms increments from a holding potential of −50 mV (Figure 3C). The normalized peak current was plotted vs. the pre-pulse duration, showing the recovery time constant from the inactivation was 136.8 ms fitted by a single exponential equation (*n* = 20 cells in seven rats; Figure 3D). We also investigated the time to peak of the T-type currents (Figure 3E), which was varied from 6 ms to 43 ms, with a mean latency of 19.8 ± 2.5 ms (*n* = 20 cells in seven rats) at the duration of 1,000 ms. Next, we asked whether there was some relationship between the amplitude and the time to peak of T-type current. Figure 3E showed that the relationship index was −0.39 (Pearson Correlation Coefficient), indicating a low negative correlation. Together, these results suggested that the peak amplitude of T-type currents was variable from cell to cell and depended on the duration of hyperpolarization pre-pulse.

**Figure 4:**
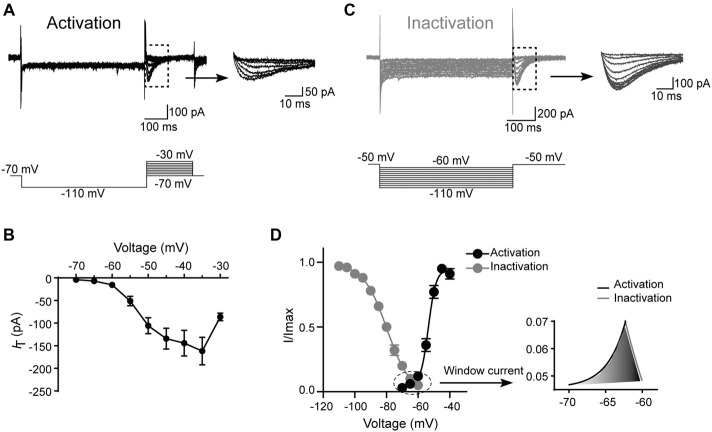
Kinetic properties of T-type current in SG neurons. **(A)** Top left: traces represent families of T-type currents evoked in a representative SG neuron. Top right: enlargement of the T-type currents in the rectangle shown on the left. Bottom is the voltage-clamp protocol used to elicit currents. **(B)** Activation current-voltage relationship of the T-type current from the same neurons shown in panel **(A)**. **(C)** Top left: traces represent families of averaged T-type currents evoked in a SG neuron. Top right: enlargement of the T-type currents in the rectangle shown on the left. Bottom is the voltage-clamp protocol used to elicit currents. **(D)** The average T-type current activation and steady-state inactivation curves. Right panel is the enlargement of window current in the circle shown on the left. Activation curve was fitted with the following Boltzmann sigmoid equation: *I*/*I*_max_ = 1/[1 + exp[(*V*_0.5_ – *V*)/*k*]], in which *I* is the amplitude of current at step potential, *I*_max_ represents the maximal current amplitude, *V*_0.5_ is the midpoint potential, *V* is the membrane potential, and *k* is the slope factor. Inactivation curve was fitted with the following Boltzmann equation: *I*/*I*_max_ = 1–1/[1 + exp[(*V − V*_0.5_)/*k*]], in which *I* is the amplitude of current at step potential, *I*_max_ represents the maximal current amplitude, *V*_0.5_ is the midpoint potential, *V* is the membrane potential, and *k* is the slope factor.

Next, the kinetics of T-type currents in SG neurons were studied. The channels were deinactivated for 500 ms at the holding potential of −110 mV before stepping to a family of voltages (from −70, to −30 mV, in a 5-mV step) for 200 ms to activate the T-type channels (Figure 4A). The average current–voltage curves were constructed and indicated that T-type current threshold was −70 mV and reached maximal amplitude at about −35 mV (Figure 4B). To determine the activation properties, the steady–state activation curves were traced by plotting the normalized peak currents vs. command voltage steps and fitted the plots by the Boltzmann equation (Figure 4D). The activation curve showed strong voltage dependence with a half-activation potential (V_0.5_) of −53.5 ± 0.3 mV and a slope factor of 3.1 ± 0.2 mV (*n* = 17 cells in eight rats).

To study the steady-state inactivation of T-type current, the membrane potential was held at −50 mV, and the channels were deinactivated for 500 ms from −60 mV to −110 mV (in a 5-mV step) before stepping to −50 mV to activate the channels (Figure 4C). Steady-state inactivation of the T-type current was estimated by fitting the relative amplitude of the current as a function of the hyperpolarizing pre-pulse with the Boltzmann equation (Figure 4D). The mean half-steady-state inactivation potential (V_0.5_) was −79.7 ± 0.3 mV and the slope factor was 7.4 ± 0.3 mV (*n* = 31 cells in 10 rats). Thus, the T-type calcium current was fully deinactivated at a holding potential negative to −110 mV and was completely inactivated at the potentials near −60 mV. The overlap region between the activation and inactivation curves (“window” current) was shown in Figure 4D, right. Interestingly, the window current which displayed tonic activity at potentials close to the resting membrane potential (RMP) was small in SG neurons, indicating that these channels are relatively inactive at rest and may require a hyperpolarizing stimulus to deinactivate and recruit more T-type channels for burst firing.

### Intrinsic Membrane Properties of SG Neurons Expressing or Lacking T-Type Currents

Since our above and other electrophysiological results showed that T-type currents cannot be detected in all the SG neurons, we are curious about what types of SG neurons express T-type currents. This is important because the primary nociceptive afferents terminate with a specific distribution pattern onto superficial SDH neurons. Moreover, all of the neurons in lamina II are interneurons (Todd, 2010), including excitatory and inhibitory interneurons. To establish the link between the electrophysiological function and the morphological feature of the T-type channel-expressing neurons, we performed both voltage- and current-clamp experiments in K^+^-based intracellular solutions containing neurobiotin 488 on 47 neurons from 42 parasagittal spinal cord slices (*N* = 30 rats).

**Figure 5:**
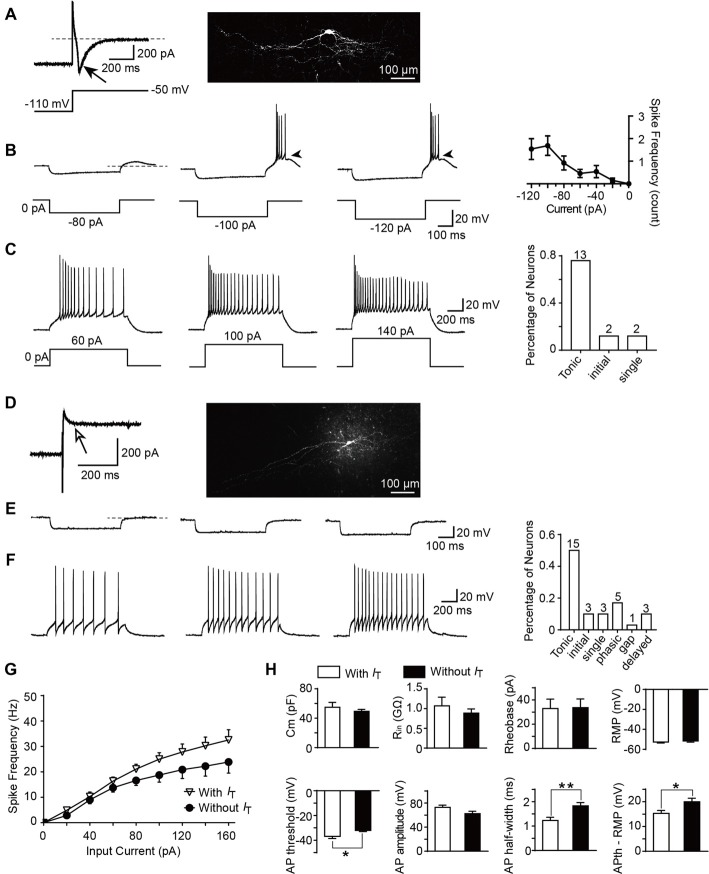
Active and passive membrane properties of T-type current-expressing and -lacking SG neurons. **(A)** Left: a representative T-type current trace evoked by 1 s hyperpolarizing potential (−110 mV) to test potential (−50 mV). Right: morphology of the neurobiotin 488-filled neuron that shown on the left. Black arrow indicates T-type current. **(B)** Representative current-clamp traces from the same SG neuron in **(A)** at the indicated hyperpolarizing currents of −80 pA, −100 pA and −120 pA. Right panel: plot of rebound depolarization-evoked spike frequency as a function of test potentials. Arrowheads indicate low threshold spikes. **(C)** Representative current-clamp traces from the same SG neuron in **(A)** depicting active membrane responses to increasing depolarizing current injection of 60 pA, 100 pA and 140 pA. Right panel: the fraction of various firing pattern in T-type calcium channel-expressing neurons (the numbers indicate the neurons examined). **(D)** Left: a representative response to the same protocol in **(A)** in a neuron lacking T-type calcium channel. Right: morphology of the neurobiotin-filled neuron that shown on the left. White arrow indicates no detection of T-type current. **(E)** Representative current-clamp responses to the same protocol in **(B)** from the same SG neuron in **(D)**. **(F)** Representative current-clamp traces from the same SG neuron in **(D)** depicting active membrane responses to the same protocol in **(C)**. Right panel: The fraction of various firing pattern in T-type current-lacking neurons (the numbers indicate the neurons examined). **(G)** Corresponding average active spikes for tonic firing neurons in T-type current-expressing and -lacking neurons. **(H)** Bar plots showing the various intrinsic membrane properties of SG neurons expressing or lacking T-type current. Cm: membrane capacitance. R_in_: input resistance. Rheobase: rheobase current. RMP: resting membrane potential. AP: action potential. APth: action potential threshold. *P < 0.05, **P < 0.01.

To determine whether T-type currents are expressed, the neurons were activated by a hyperpolarizing step voltage pulse from the holding potential of −50 mV to −110 mV for 1 s followed by a depolarization to −50 mV for 1 s. We defined T-type current expressed in a neuron if the peak amplitude of the inward current is more than 6 pA (corresponding to the minimum current we recorded in the transverse slices) after 15-min co-application of TTX and ZD7288 (10 μM). ZD7288 is a specific HCN channel blocker which was used to exclude the possible contamination of tail current of HCN channel (Supplementary Figure S1). Among all the neurons examined from parasagittal slices for morphological experiments, the number of SG neurons which express or lack T-type currents were 17 and 30, respectively. Figure 5A showed a classic T-type current and the corresponding cellular morphology (classified as vertical neuron) from a representative SG neuron. Given that T-type currents often lead to rebound depolarization following the injection of hyperpolarizing currents (Zhang et al., 2017), we next examined the features of the rebound depolarization in neurons expressing T-type currents. Among the 17 neurons expressed T-type currents, rebound depolarization-induced spike (low-threshold spike, LTS) was observed in 13 neurons. Subthreshold rebound depolarization (without spike) was observed in two neurons (Supplementary Figure S2). In the left two neurons, spontaneous Na ^+^-dependent spikes were detected (Supplementary Figure S2). Since we cannot determine whether the spike is an LTS or a spontaneous one, the spikes of the latter two neurons were not included in the analysis. As shown in Figure 5B, the frequency of LTS depended on the tested hyperpolarizing current. Averaged spike frequencies vs. the hyperpolarizing pre-pulses were shown in the right panel (*n* = 13 cells in nine rats). Meanwhile, we found that the mean latency to peak of the rebound depolarization was 69.6 ± 19.3 ms at −120 pA. Our previous study has shown that SG neurons exhibit various firing patterns, encompassing tonic firing, delayed firing, single spike, initial burst, phasic firing, gap firing and reluctant firing (Hu et al., 2016). Exemplars of tonic firing at different depolarized membrane potentials were shown in Figure 5C. In T-type channel-expressing neurons, 13 out of 17 were tonic firing (76.5%; Figure 5C, right). In contrast, in those neurons lacking T-type currents (Figure 5D), no rebound depolarization was detected (Figure 5E), though they may display as a tonic firing pattern (15 out of 30 neurons, 50%) responding to the depolarized currents (Figure 5F). Moreover, in tonic firing neurons expressing T-type current or not, the frequencies of Na ^+^-dependent spikes were compared (Figure 5G). Strong depolarizing currents (≥80 pA) caused a greater frequency of spikes in neurons expressing T-type current (*n* = 11 cells in eight rats) compared to those lacking it (*n* = 8 cells in eight rats). However, this did not reach a significant difference (the neurons with spontaneous firing were not included in the analysis).

Since passive membrane properties determine the voltage responses of neurons, we next investigated whether these properties were different between the two neuronal types. As shown in Figure 5H and Table 1, we found that, between those neurons expressing T-type current or not, there were no significant differences for the membrane capacitance (Cm), RMP, input resistance (Rin), rheobase current and action potential (AP) amplitude. However, the AP threshold was more negative in neurons expressing T-type current than those lacking it (*p* = 0.017; SG neurons with *I*_T_; *n* = 17 cells in 12 rats; SG neurons without *I*_T_; *n* = 30 cells in 18 rats), suggesting *I*_T_-expressing neurons had more excitable membranes. Consistently, the different values of AP threshold and RMP were smaller in *I*_T_-expressing neurons than those lacking *I*_T_ (*p* = 0.016), suggesting the former neurons were easier to be excited to stimulus. In addition, neurons expressing T-type currents showed shorter half-width of AP than those lacking it (*p* = 0.004; SG neurons with *I*_T_; *n* = 17 cells in 12 rats; SG neurons without *I*_T_; *n* = 30 cells in 18 rats).

**Table 1:** Membrane properties of T-type current-expressing or -lacking neurons.

Parameter	Group	Total	Number of cells
Capacitance (pF)	With *I*_T_	55.92 ± 6.98	17
	Without *I*_T_	49.35 ± 3.34	30
Input resistance (GΩ)	With *I*_T_	1.17 ± 0.23	17
	Without *I*_T_	0.84 ± 0.11	30
Rheobase current (pA)	With *I*_T_	32.94 ± 7.71	17
	Without *I*_T_	33.67 ± 7.12	30
RMP (mV)	With *I*_T_	−51.24 ± 1.21	17
	Without *I*_T_	−52.27 ± 1.15	30
AP threshold (mV)	With *I*_T_	−36.94 ± 1.78*	17
	Without *I*_T_	−32.40 ± 0.94	30
AP amplitude (mV)	With *I*_T_	68.80 ± 3.83	17
	Without *I*_T_	60.36 ± 3.23	30
AP half-width (ms)	With *I*_T_	1.19 ± 0.12**	17
	Without *I*_T_	1.78 ± 0.13	30
APth − RMP (mV)	With *I*_T_	14.29 ± 1.59*	17
	Without *I*_T_	19.87 ± 1.42	30

Values given are the means ± SEM. *I*_T_ indicates T-type current. *P < 0.05; **P < 0.01.

### Cell-Type Specific Expression of T-Type Current in Various Morphological Patterns of SG Neurons

**Figure 6:**
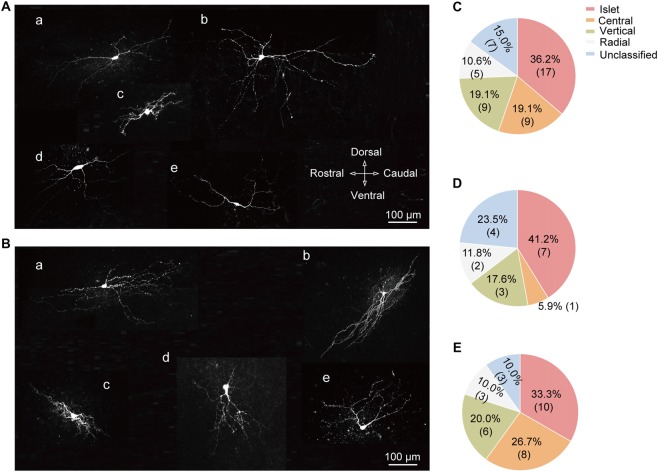
Cell-type specific expression of T-type currents in SG neurons. **(A)** Exemplar confocal images showing the neurobiotin 488-filled T-type current-expressing neurons. They were classified as: islet cell **(a)**; radial cell **(b)**; central cell **(c)**; vertical cell **(d)**; and unclassified cell **(e)**. Scale bar = 100 μm. **(B)** Exemplar confocal images showing the neurobiotin 488-filled T-type current-lacking SG neurons assigned to the same order in **(A)**. **(C)** Prevalence of five different morphological subtypes in SG neurons. **(D,E)** Summary data showing the percentage of different morphological subtypes in T-type current-expressing **(D)** and -lacking *neurons* **(E)**.

Based on the previous study (Yasaka et al., 2010), SG neurons can be categorized into various subtypes according to their morphology, including the following five groups: islet, central, vertical, radial, and unclassified. Figures 6A,B illustrated the representative neuronal morphology of *I*_T_-expressing (A) and -lacking (B) neurons. The proportion of each type of SG neurons was illustrated in Figure 6C. Among all the neurons examined, the highest proportion of SG neurons was islet (36.2%). Furthermore, we found that the distributions of SG neurons expressing T-type current or not were distinct in various subgroups. Islet neurons were the highest proportion in both SG neurons with or without *I*_T_ (41.2% and 33.3%, respectively; Figures 6D,E). Next, we set out to test whether there are some correlations between T-type current density and morphological parameters, such as soma diameter and dendritic length that elongated at the rostrocaudal or dorsoventral direction which determine the morphological type of SG neurons (Yasaka et al., 2007). As shown in Supplementary Figure S3, the values of Pearson’s r were 0.17, 0.30 and −0.31, respectively. These results indicated that the relationship between T-type current density and morphological parameters were not quite correlated. Together, these results revealed a specific expression pattern of T-type channel in different morphological SG neurons.

### Effect of the T-Type Calcium Channel Blocker on Electrophysiological Properties of SG Neurons

**Figure 7:**
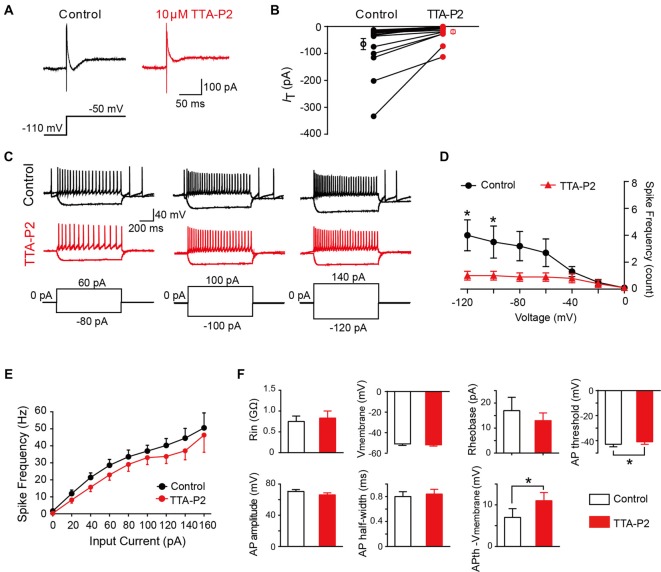
Effect of T-type calcium channel blockers on the intrinsic membrane properties of SG neurons. **(A)** Representative T-type current traces in response to voltage-clamp protocol shown below traces in the treatment of control (black) and TTA-P2 (red). **(B)** Population data showing the peak amplitude of T-type currents in the absence (black) and presence of TTA-P2 (red). **(C)** Examples of depolarizing and hyperpolarizing traces in response to current-clamp protocols shown below traces before (black) and after (red) the treatment of TTA-P2. **(D)** Population data showing the rebound depolarization-evoked spike frequency plotted as a function of test potentials in the absence (black) and presence of TTA-P2 (red). **(E)** Population data showing the AP spike frequency plotted as a function of elicited currents in the absence (black) and presence of TTA-P2 (red). **(F)** Bar plots showing the various intrinsic membrane properties of SG neurons in the treatment of control (black) and TTA-P2 (red). V_membrane_: membrane potential measured at I_hold_ = 0. *P < 0.05.

To investigate whether the differences in intrinsic membrane properties between T-type-expressing and -lacking neurons were due to the function of T-Type calcium channels (Figure 5), we next performed a series of similar experiments in the presence of a specific T-Type channel blocker TTA-P2 (Choe et al., 2011). As shown in Figures 7A,B, we observed that 10 μM TTA-P2 significantly decreased the T-type calcium current amplitude to 27 ± 5% of control (65 ± 20 pA; *p* = 0.008; *n* = 17 cells in 10 rats). Previous studies have suggested that the LTS is attributed to the activation of T-type channels; therefore, we next studied the effect of TTA-P2 on the LTS and found a significant decrease in the frequency of LTS by TTA-P2 treatment (Figures 7C,D). However, TTA-P2 did not affect the frequency of spikes elicited by a series of depolarizing currents (Figures 7C,E). Furthermore, it is interesting to see that the treatment of TTA-P2 significantly increased the AP threshold (*p* = 0.041) and the different values of AP threshold and membrane potential (I_hold_ = 0; *p* = 0.018). Other membrane properties, such as membrane potential (I_hold_ = 0), Rin, Rheobase current, AP amplitude and AP half-width were not significantly affected by TTA-P2 (Figure 7F). These results suggested that T-Type calcium channels play a role in regulating intrinsic membrane properties of SG neurons.

## Discussion

Since 2003, genetic linkage studies have implicated T-type channels are involved in the mechanism of pathological pain (Kim et al., 2003; Choi et al., 2007, 2016; Na et al., 2008). In addition, upregulation of T-type channels in SDH has been found in a variety of animal pain models, suggesting a crucial role of spinal T-type channels in chronic pain (Stemkowski et al., 2016; Lai et al., 2017; Li et al., 2017). However, the specific cellular distribution and function of T-type channels in SDH remain unclear. In this study, we provided the first direct evidence for the cell-type specific distribution of T-type channels in SG neurons. Moreover, we identified that blockade of T-type currents may decrease the excitability of SG neurons.

### Pharmacological Blockade of T-Type Channels

T-type channels are the important target of pain therapeutics (François et al., 2014; Bourinet et al., 2016). For example, T-type channel blocker ethosuximide applied by i.p., i.t., or directly onto the spinal cord could reverse the pathological pain (Matthews and Dickenson, 2001; Flatters and Bennett, 2004; Chen et al., 2015). Besides ethosuximide, Zonisamide and Z944 also have been recruited in the preclinical trial for pain treatment (Zamponi, 2016). Up to now, Ni^2+^ is still the most often used T-type channel blockers for animal experiments, possibly due to its high efficiency and reversible action (Chen et al., 2015; Voisin et al., 2016; Zhang et al., 2017). We observed that NiCl_2_ (0.2 mM) decreased the amplitude of T-type calcium current to 29.6 ± 4.6% of control in SG neurons, which was lower than that of the observation in lamina II–V SDH neurons (Ryu and Randic, 1990), possibly due to the different holding potential (−90 mV in Ryu’s study vs. −110 mV in our study) used. The holding potential-dependent modulation of T-type current was also observed for TTA-A2 (Francois et al., 2013), suggesting that the binding of those drugs to T-type channel was different between resting channels and inactivated channels.

Recently, Tat-3.2-III–IV, a cell-penetrating Tat peptide, was found to be able to competitively disrupt the interaction between USP5 and the Cav3.2 channel, resulting in the reversal of pathological pain (Garcia-Caballero et al., 2014; Gadotti et al., 2015; Stemkowski et al., 2016, 2017). These results provide a new concept for the development of pharmacological target for T-type channel trafficking by the post-translational modification rather than current activity.

### Kinetic Properties of T-Type Channels in SG Neurons

T-type channels are unique among the voltage-gated calcium channels for their fast kinetics and low voltages of activation and inactivation, which allows them to open at voltages near RMP of most neurons. In consistent with previous studies (Ryu and Randic, 1990; Ku and Schneider, 2011), our data showed that the T-type currents in SG neurons were activated at around −70 mV, reaching a maximum at −35 mV. V_0.5_ for activation and inactivation potentials in HEK-293 cell with stably expressed *α1G*, *α1H* and *α1I* were −43.8 mV to −45.5 mV and −72.0 mV to −72.8 mV, respectively (Klökner et al., 1999). In another study of these cloned channels, the V_0.5_ values were −41.5 mV to −49.3 mV and −69.8 mV to −74.2 mV (Chemin et al., 2002). In this work, we observed the V_0.5_ for activation and inactivation were −53.5 mV and −79.7 mV, respectively. Due to the lack of isoform-specific pharmacological tool, we are not able to distinguish the isoforms of the T-type current. An alternative method is to perform single-cell RT-PCR after each electrophysiological recording. However, the low detection rate of Cav3 mRNA (12/29) by single-cell RT-PCR indicated that some of the T-type channels might be located in dendrites instead of soma (Ku and Schneider, 2011). The expression and function of T-type channels in dendritic trees need to be further investigated.

The amplitude of T-type current in SG neurons we recorded (6.1 pA to 415.4 pA) is lower than that has been reported in spinal lamina II–V neurons (50 pA to 1 nA; Ryu and Randic, 1990). Moreover, the current density in this study is also lower than that of superficial SDH neurons (6 pA/pF; Ku and Schneider, 2011). These differences might be due to the different extracelluar solutions they used which contain 2–5 mM Ba^2+^. Ba^2+^ has been used as the charge carrier of T-type channel in a number of studies (Huguenard, 1996). However, the impact of Ba^2+^ on the characteristic properties of the expressed currents should be taken into consideration (McDonald et al., 1994). Therefore, the recording conditions should be noted when the kinetics of T-type current are compared among various reports. In this study, we used the physiological concentration of Ca^2+^ without using of any charge carrier. In aggregate, the kinetic features of T-type channels in SG neurons enable them to modulate the neuronal excitability at rest.

### Specific Cellular Distribution of T-Type Currents in SG Neurons

T-type channels belong to the voltage-gated calcium channel family, consisting of three isoforms: Cav3.1 (*α1G*), Cav3.2 (*α1H*) and Cav3.3 (*α1I*; Senatore et al., 2014). *In situ* hybridization studies have demonstrated that *α1G*, *α1H* and *α1I* were present in SDH (Talley et al., 1999). Although existing data imply the involvement of spinal T-type channel in nociceptive regulation, its cellular distribution remains controversial (Ku and Schneider, 2011; Hughes et al., 2012). Therefore, it will be interesting to see what types of SG neurons express T-type channels.

Intracellular recording from superficial SDH neurons of 9–18-day-old SD rats found that a low-threshold Ca^2+^-dependent response could be activated at membrane potentials more negative than −65 mV (Murase and Randić, 1983). In another pioneer study, LVA calcium currents were recorded in 64 out of 89 voltage-clamped lamina II–V neurons of the SDH in 2–4-week-old SD rats (Ryu and Randic, 1990). However, Ku et al. found that nearly all the dorsal horn neurons express T-type currents in 9–14-day-old hamsters, and the expressions of T-type currents are similar in the superficial and deep dorsal horn (Ku and Schneider, 2011). The above studies suggested that the expression of T-type channels in dorsal horn neurons may exhibit species and developmental differences. In this study, we performed both voltage- and current-clamp recordings in transverse and parasagittal spinal cord slices from 3-week to 5-week-old SD rats. We found that the fractions of T-type current-expressing neuron are 44.5% and 39.1% in transverse and parasagittal slices, respectively, with a total proportion of 43.5%. These incidences seem to be higher than that of mice, which is about 20% (Graham et al., 2007; Walsh et al., 2009; Tadros et al., 2012). Moreover, the incidence of T-type channel-expressing neuron in our study is much lower than that has been reported in 6–10-week-old Wistar rats (Yasaka et al., 2010). They found that the incidence of a transient inward current which is probably mediated by low threshold calcium channel was about 10% in excitatory neurons and 80% in inhibitory neurons (Yasaka et al., 2010). This different incidence may cause by the usage of the K^+^-based intracellular solution in in Yasaka et al.’s (2010) work. Moreover, most of the neurons (more than 50%) in SDH co-expressed *I*_h_ and *I*_T_ (Hughes et al., 2012). Given that the tail current in *I*_h_-expressing neurons resembled a T-type current (Supplementary Figure S2), we mainly used Cs^+^-based intracellular solution to study *I*_T_ except for the observation in parasagittal slices, in which K^+^-based intracellular solution is more proper to study the intrinsic membrane features. In addition, in the latter condition, HCN channel antagonist ZD7288 was used to help decrease the impact of tail current on *I*_T_.

An interesting study showed that, the prevalence of T-type current in random selected lamina II–III neurons and parvalbumin-expressing neurons were around 20% and 40%, respectively (Hughes et al., 2012). This raises the possibility that the T-type currents might exhibit a specific expression in subtypes of SG neurons. Different firing patterns have been found in SG neurons (Ruscheweyh and Sandkuhler, 2002; Cui et al., 2011; Tadros et al., 2012). This work reported that the percentage of tonic firing neurons was 60.9% in all the SG neurons, which is close to the fraction (62%) in adult rats (Yasaka et al., 2010). Meanwhile, we found that in T-type channel-expressing or -lacking neurons, the fractions of tonic firing neurons are 79.4% and 50.9%, respectively. Given that most of the tonic firing SG neurons (87%) have been demonstrated to be inhibitory neurons (Yasaka et al., 2010), T-type current-expressing SG neurons we observed might mainly be the inhibitory neurons. Another classification method for SG neurons is based on their morphology (Grudt and Perl, 2002; Maxwell et al., 2007; Yasaka et al., 2007, 2010). According to the dendritic arborizations, SG neurons can be classified into five groups: islet, central, vertical, radial, and unclassified neurons. Due to the reason that most of the dendritic trees of lamina II neurons are elongated in the rostrocaudal axis, parasagittal slices were used in our study to reconstruct the SG neurons. In connection with our observation of T-type current and firing pattern, 41.2% of the T-type currents-expressing neurons were found to be islet cells, which has been indicated to be the inhibitory cells (Maxwell et al., 2007). However, we did not find any close correlation between T-type current density and morphological parameters. Together, both the electrophysiological and morphological features supported the hypothesis that most of the T-type current expressing neurons are inhibitory neurons.

### Functional Role of T-Type Channels in SG Neurons

Though the modulation of T-type channels plays an important role in the animal model of pathological pain, the physiological significance of Cav3 channels in SG neurons has not been well established yet. The most prominent functional role of T-type channels is to promote the generation of LTS, which can lead to bursting firing in neurons. Our data showed that LTS was recorded in most of the T-type current-expressing SG neurons whose firing patterns are tonic. However, tonic firing neurons were also observed in T-type current-lacking neurons. These suggested that the T-type current might play a role in part of the tonic-firing neurons. Other ionic conductance involved in tonic firing might include voltage-gated Na^+^ and K^+^ currents (Melnick et al., 2004). Moreover, in consistent with the previous observations in deep cerebellar nuclear neurons (DCNs; Boehme et al., 2011) and thalamic reticular neurons (Chausson et al., 2013), we found that TTA-P2 dramatically blocked the LTS. However, we found that TTA-P2 did not affect the frequency of depolarization-induced spike, which is in line with the reports showing that TTA-P2 did not change the firing frequency of DCNs (Boehme et al., 2011) or ventrobasal nucleus of thalamocortical neurons (Dreyfus et al., 2010).

Besides the regulation of spike frequency, several additional functional roles for T-type currents have been identified. These include the regulation of intrinsic neuronal excitability, modulation of synaptic release, and boosting of synaptic signals. We found that the AP threshold was more negative in *I*_T_-expressing neurons than those lacking it, indicating a possible role of *I*_T_ in modulating intrinsic neuronal excitability. Indeed, application of TTA-P2 in our study changed the values of AP threshold toward the positive direction. Meanwhile, the difference between AP threshold and membrane potential is larger than TTA-P2 treatment. Similar result has been reported in DRG neurons, in which TTA-A2 increased the AP threshold (Francois et al., 2013). These data, together with the demonstration that TTA-P2 inhibited the LTS in SG neurons, indicate that T-type channels play a substantial role in the modulation of neuron excitability.

In this study, 43.5% of the SG neurons express T-type channels, which might be involved in the integration and processing of nociceptive information. Furthermore, given the existence of “window current”, small range of Cav3 channels are activated near RMP, leading to a tonic calcium influx, which results in the control of some intracellular Ca^2+^-dependent processes, such as presynaptic neurotransmitter release. This is further supported by the evidence that T-type channel antagonist TTA-P2, as well as Ni^2+^ decreased the frequency of mEPSC in spinal lamina I and II neurons (Jacus et al., 2012; Garcia-Caballero et al., 2014). However, TTA-A2 had no effect either on the mIPSC of the lamina I and II neurons or the mEPSC of the lamina III–VI neurons (Jacus et al., 2012). These results suggested that the physiological function of T-type channels in regulating presynaptic neurotransmitter release and somatic T-type current are cell-type-dependent. Therefore, it is very necessary to further study the subcellular distribution of T-type calcium channels in SDH neurons.

In summary, our study revealed a specific distribution pattern of T-type calcium channels in various SG neuron types. The majority types of T-type current-expressing neurons exhibit the tonic firing pattern and islet morphology. Furthermore, the excitability is higher in IT-expressing neurons than in IT-lacking neurons, which could be imitated by a specific T-type calcium channel blocker. These findings could help to elucidate the physiological function of Cav3 T-type calcium channels in sensory signaling.

## Author Contributions

TL conceptualized the study, interpreted the data, drafted and edited the manuscript. JW and SP performed the morphological and electrophysiological experiments. LX, XC, HK, MZ, DZ and CJ assisted in the analysis of morphological and electrophysiological data. All authors approved the final version of the manuscript and agreed to be accountable for all aspects of the work. All persons designated as authors qualify for authorship, and all those who qualify for authorship are listed.

## Conflict of Interest Statement

The authors declare that the research was conducted in the absence of any commercial or financial relationships that could be construed as a potential conflict of interest.
