# Supplementary material for: Cell-Type Specific Distribution of T-Type Calcium Currents in Lamina II Neurons of the Rat Spinal Cord
Source: Front Cell Neurosci. 2018 Oct 17;12:370. doi: 10.3389/fncel.2018.00370 (PMC6199353; doi:10.3389/fncel.2018.00370)
Supplement: Supplementary file 1 [file Data_Sheet_1.docx]

Supplementary Material

Cell type-specific distribution of T-type calcium currents in lamina II neurons of the rat spinal cord

**Jing Wu^1†^, Sicong Peng^1†^, Linghui Xiao^1^, Xiaoe Cheng^2^, Haixia Kuang^1^, Mengye Zhu^3^, Daying Zhang ^3^, Changyu Jiang ^4^, AND Tao Liu ^1,4,5*^**

**^1^***Department of Pediatrics, the First Affiliated Hospital of Nanchang University, Nanchang, China,* **^2^** *Department of Anesthesiology, the First Affiliated Hospital of Nanchang University, Nanchang, China,* **^3^***Department of Pain Clinic, the First Affiliated Hospital of Nanchang University, Nanchang, China,* **^4^***Jisheng Han Academician Workstation for Pain Medicine, Nanshan Hospital, Shenzhen China,* **^5^***Center for Experimental Medicine, the First Affiliated Hospital of Nanchang University, Nanchang, China*

*** Correspondence:
Tao Liu**
liutao1241@ncu.edu.cn

†These authors contributed equally to this work.


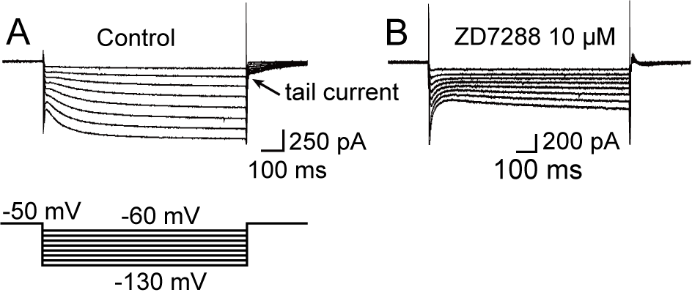


**Figure S1.** Effect of HCN channel antagonist on the tail current. Representative current responses to hyperpolarization voltage steps in the absence **(control, A)** and presence of ZD7288 **(B)**. Lower panel shows the voltage-clamp protocol. Arrow marks the tail current.


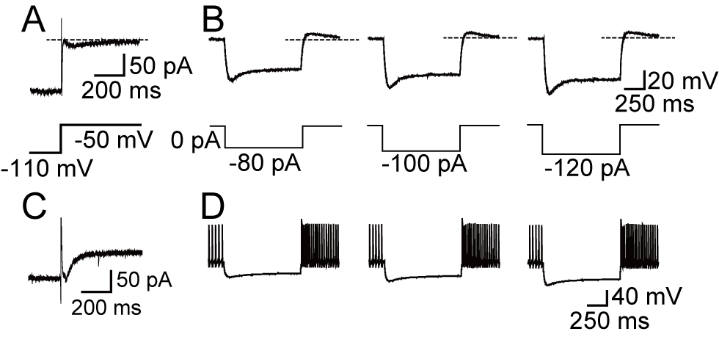


**Figure S2.** Responses of SG neurons to hyperpolarization voltage and current stimulation. **(A-B)** Representative traces of T-type current (A) and subthreshold rebound depolarization (B) from the same neuron. Lower panels show the voltage- or current-clamp protocols. **(C-D)** Representative traces of T-type current (C) and spontaneous Na+-dependent spikes (D) from the same neuron.


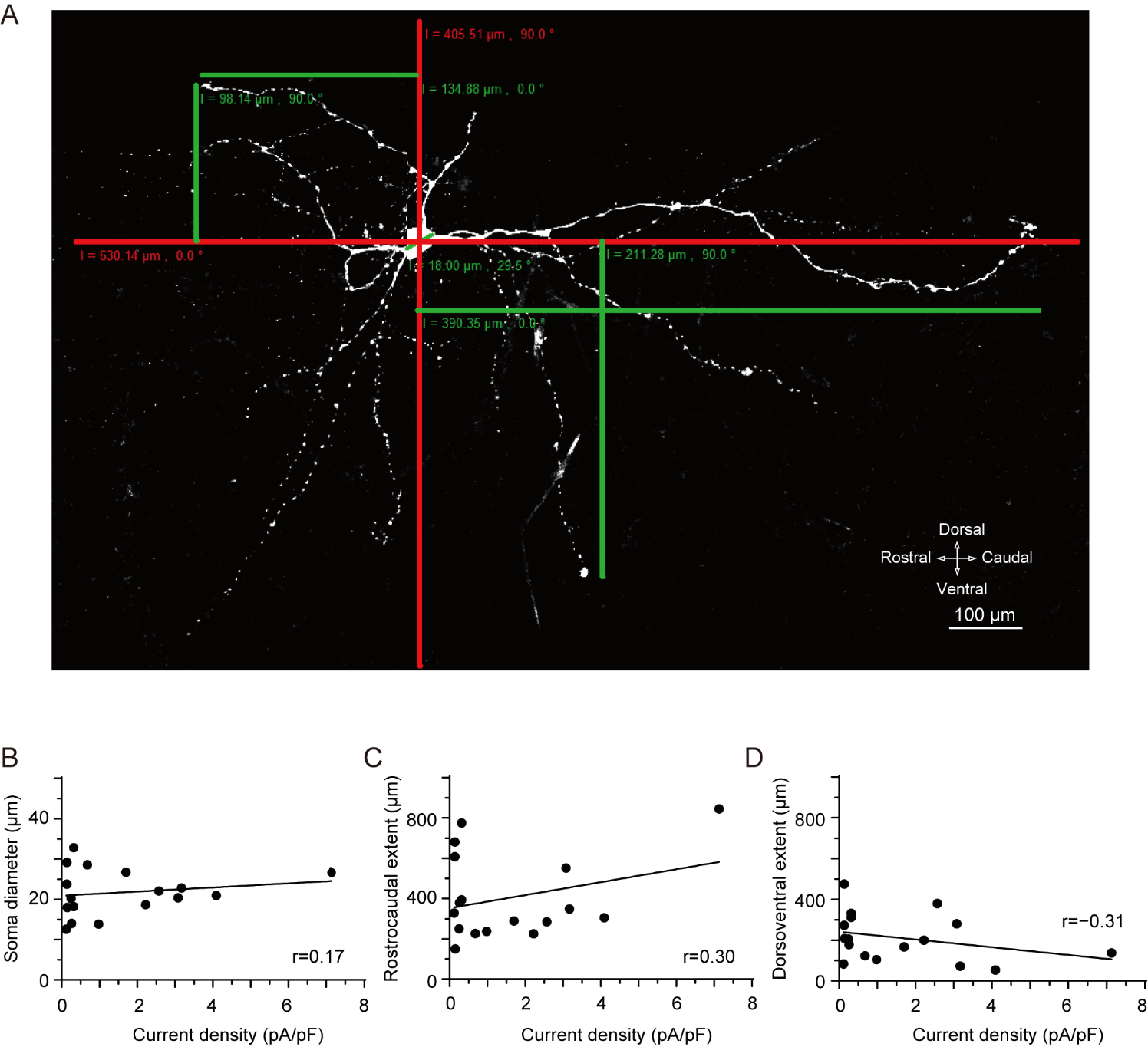


**Figure S3.** Pearson correlations comparing T-type current density and morphological parameters. **(A)** Representative diagram showing the parameters measured. **(B)** Pearson correlation scatter plot of T-type calcium current density and soma diameter. **(C-D)** Pearson correlation scatter plot of T-type calcium current density and dendritic length elongated at the rostrocaudal or dorsoventral direction.
